# Independent relationship between serum ferritin levels and dyslipidemia in Chinese adults: A population study

**DOI:** 10.1371/journal.pone.0190310

**Published:** 2017-12-22

**Authors:** Jiang Li, Weimin Bao, Tie Zhang, Yun Zhou, Hui Yang, Hongbing Jia, Rui Wang, Yongtong Cao, Cheng Xiao

**Affiliations:** 1 Department of Laboratory Medicine, China-Japan Friendship Hospital, Beijing, China; 2 Community Health Service Center of Hepingli of Dongcheng District, Beijing, China; 3 Blood Screening Laboratory, Beijing Red Cross Blood Center, Beijing, China; 4 Institute of Clinical Medicine, China-Japan Friendship Hospital, Beijing, China; Shanghai Diabetes Institute, CHINA

## Abstract

**Objective:**

Several studies have indicated that elevated levels of circulating ferritin are associated with disturbances in energy metabolism. But none of this gave a clearly pathologic mechanism. We aimed to explore the independent relationship between serum ferritin levels and dyslipidemia.

**Methods:**

We performed multivariable logistic regression analyses to estimate the odds ratios (ORs) for dyslipidemia, lipid parameters, the homeostasis model assessment of insulin resistance (HOMA-IR) and the risk of diabetes, according to sex-specific quartiles of serum ferritin by using the data of China Health and Nutrition Survey (2009 CHNS). We used three models to estimate the strength of the correlation. The basic model (Model 1) is without adjustment and the Model 2 and Model 3 are adjusted for demographic, anthropometric, and lifestyle confounding factors.

**Results:**

In both genders, the ORs for high TG level, TC level and LDL-C level increased progressively and for HDL-C decreased across the ferritin quartiles (P<0.001 for trend). After adjustment for confounding factors in different logistic regression models, the results remained unchanged. The ORs for the risk of diabetes and high HOMA-IR level in the highest quartile group of serum ferritin levels were significantly increased in Model 1, but after adjustment for lipid parameters, the ORs for the risk of diabetes was decreased from 1.91 (95% CI: 1.37–2.67; P<0.001 for trend) to 1.48 (95% CI: 1.03–2.12; P = 0.036 for trend) in men, and from 5.40 (95% CI: 3.38–8.63; P<0.001 for trend) to 1.43 (95% CI: 0.83–2.43; P = 0.498 for trend) in women, and the ORs for IR was decreased from 1.86 (95% CI: 1.57–2.20; P<0.001 for trend) to 1.25 (95% CI: 1.05–1.50; P = 0.114 for trend) in men, and from 1.93 (95% CI: 1.63–2.28; P<0.001 for trend) to 1.24 (95% CI: 1.01–1.51; P = 0.012 for trend) in women.

**Conclusion:**

Our results provide evidence that serum ferritin levels are significantly associated with lipid parameters, independent of glucose metabolism disorders and components of metabolic syndrome (MetS). Thus, serum ferritin plays a key role in energy metabolism disorders and may affect glucose metabolism through lipid metabolism.

## Introduction

Dyslipidemia is a condition in which the amount of lipids is abnormal. With economic development and lifestyle changes, elevated serum lipids have become more common in China[1]. Dyslipidemia is an important component of metabolic syndrome (MetS) and a well established risk factor for cardiovascular diseases (CVDs)[2]. It is associated with obesity, hypertension, type 2 diabetes (T2DM) and other disturbances.

Ferritin is not only used as a clinical biomarker to evaluate iron status, but plays an important role in energy metabolism disorders. In our previous study[3], we have confirmed that there was a positive association between higher ferritin levels and the prevalence of MetS and its components, particularly hypertriglyceridemia and hyperglycemia. Some other studies showed that the association between serum ferritin and disrupted glucose metabolism became weak or non-significant after further adjustment for lipid parameters or components of MetS in logistic regression model (Table 1).

**Table 1:** Effect estimates of type 2 diabetes according to ferritin levels in different logistic regression models in previous studies.

Source	Model	Comparison and effect estimates (95% CI)		Adjustment for covariates
		Men	Women	
Jehn et al, 2007[7]	Model 1	1.51(0.98–2.31)		Age, center, ethnicity, smoking, alcohol, BMI
	Model 2	0.79(0.48–1.32)		Model 1+**HDL-C**, **waist circumference, hypertension, fasting glucose level, fasting TG level**, fasting insulin level, inflammation score
Kim et al, 2011[10]	Model 1	1.71(1.38–2.12)	1.50(1.05–2.13)	Age
	Model 2	1.42(1.14–1.78)	1.30(0.89–1.89)	Model 1+ **BMI, waist circumference, systolic and diastolic blood pressure, TG, HDL-C, TC,** CRP, smoking, alcohol use and menopause status in women
Luan er al, 2008[16]	Model 1	4.34(2.31–8.14)		Age, sex
	Model 2	2.96(1.53–5.72)		Model 1+smoking, drinking, sedentary time, diabetes family history, **central obesity, high blood pressure, abnormal blood lipid**, intake of calories, fiber, high percentage of energy from fat
Akter et al, 2017[17]	Model 1	1.42(1.03–1.96)		Age, sex, month of examination, leisure time physical activity, occupational physical activity, smoking, alcohol drinking, shift work, sleep duration, family history of diabetes, hypertension, BMI
	Model 2	1.20(0.86–1.67)		Model 1+ **HDL-C, TG,** CRP, adiponectin, ALT, GGT

TC, total cholesterol; TG, triglycerides; HDL-C, high-density lipoprotein cholesterol; LDL-C, low-density lipoprotein cholesterol; HOMA-IR, homeostatic model assessment of insulin resistance; ALT, alanine aminotransferase; GGT, gamma glutamyl transpeptidase; CRP, C-reactive protein.

Glucose and lipids play important roles in energy metabolism and are regulated by the liver[2]. The interaction between glucose and lipid metabolism is complex. Recent studies have shown that dyslipidemia may disrupt glucose metabolism. The lipotoxic effects result in impaired insulin secretion and β-cell apoptosis and may contribute to the loss of β-cell function in the pathogenesis of T2DM[4].

There are few studies on the relationship between serum ferritin and dyslipidemia. So we aimed to explore the role of serum ferritin in lipid metabolism and dyslipidemia and to demonstrate that serum ferritin levels is independently associated with dyslipidemia and lipid parameters in the general Chinese adult population.

## Methods

### Ethics statement

The CHNS is an ongoing open cohort, international collaborative project between the Carolina Population Center at the University of North Carolina at Chapel Hill and the National Institute of Nutrition and Food Safety at the Chinese Center for Disease Control and Prevention. China-Japan Friendship Hospital participated in the 2009 wave of CHNS (2009 CHNS) by collecting blood samples.

All the documentation and procedures comply with Good Clinical Practice (GCP), Human Ethics Protocol Rules and related Chinese laws. The CHNS project was approved by the office of human research ethics of the University of North Carolina at Chapel Hill and the Human & Clinical Research Ethics Committee of China-Japan Friendship Hospital. All participants provided written informed consent to participate in this study. They were required to fast overnight (at least 8 hours) before blood was collected by trained phlebotomists under a standard protocol. The Institutional Review Board information can also be found at the World Wide Web site (http://www.cpc.unc.edu/projects/china).

### Study population

The CHNS started in 1989 and was designed to represent a set of large provinces with a range of economic and demographic variation. The participants of the 2009 CHNS consist of 216 communities from 9 provinces (including Heilongjiang, Liaoning, Shandong, Henan, Hubei, Hunan, Jiangsu, Guangxi, Guizhou) comprising 36 urban neighborhoods, 36 suburban neighborhoods, 36 towns and 108 villages. In this wave, blood samples were collected and tested for the first time. Details about the study design and sampling strategies are available at the World Wide Web site (http://www.cpc.unc.edu/projects/china/home.html) and elsewhere[5, 6].

In this wave of the survey, 8641 fasting blood samples from participants aged 18 and older were collected. To clarify, 252 participants who did not have serum ferritin data due to a serious hemolytic state or did not have physical examination information and 1280 participants with anemia and iron overload were excluded from the analysis. Therefore, we analyzed the data of 7109 participants aged 18 and older. We couldn’t access to information that could identify individual participants during and after data collection.

### Data collection methods

The demographic, anthropometric, and lifestyle data were collected by trained interviewers (physicians and nutritionists) through a validated questionnaire (http://www.cpc.unc.edu/projects/china/data/questionnaires). In the analysis, smoking status was grouped as never-smokers and smokers based on reported cigarette consumption. Alcohol drinking was classified based on reported consumption frequencies. Based on the standard protocol, which is similar to the National Health and Nutrition Examination Survey protocol developed by the US National Center for Health Statistics, trained interviewers measured height and weight. Height was measured to the nearest 0.1 cm, and weight in lightweight clothing was measured to the nearest 0.1 kg. BMI was calculated as weight in kg divided by height in square meters [7].

Trained phlebotomists drew fasting blood from participants’ antecubital vein in the morning under a standard protocol (http://www.cpc.unc.edu/projects/china/data/questionnaires/C09blood_Fin20090721.pdf). Blood samples were transferred to the local hospital for further treatment within 2 hours of collection. The samples were centrifuged at 3000*g* for 10 minutes at room temperature as soon as possible and separated into 9 aliquots. Except for the samples for field testing, other samples were stored in -80 degree freezers.

The fasting serum glucose measurements (enzymatic method) and routine blood examinations were performed at local hospitals. Glycated hemoglobin (HbA1c) was assessed with high-performance liquid chromatography. The calibrators and control serums were provided by the department of laboratory medicine of China-Japan Friendship Hospital and had the same lot number. Other biochemical markers were tested with an automatic clinical chemistry analyzer (Hitachi 7600 D and P models, Japan) at the department of laboratory medicine of China-Japan Friendship Hospital. Serum triglycerides (TG), total cholesterol (TC), high-density lipoprotein cholesterol (HDL-C) and low-density lipoprotein cholesterol (LDL-C) were detected using the enzymatic colorimetric method (Kyowa, Japan). High-sensitivity C-reactive protein (CRP) was measured using the immunoturbidimetric immunoassay method (Denka Seiken, Japan). The concentration of fasting serum insulin and ferritin was evaluated with a commercial radioimmunoassay kit (Beijing North Institute of Biological Technology, China).

### Dyslipidemia

Different categories of lipid parameters were defined based on 2016 Chinese guideline for management of dyslipidemia in adults[8]. Dyslipiemia was defined as TG≥1.7mmol/L, TC≥5.2mmol/L, LDL-C≥3.4mmol/L or HDL-C<1.0mmol/L.

### Diabetes and insulin resistance

Diabetes was defined as fasting glucose≥7.0mmol/L (≥126mg/dL) or a diagnosis of diabetes[9]. IR was estimated with the Homeostasis Model Assessment (HOMA-IR) equation.

### Anemia and iron overload

Anemia was defined based on the World Health Organization (WHO) hemoglobin thresholds of hemoglobin concentrations less than 130 g/L for men and less than 120 g/L for women[10]. Iron-deficiency anemia was defined as the presence of both anemia and inflammation-adjusted ferritin concentration[11] less than 15 ng/mL[12]. Iron overload was defined as inflammation-adjusted ferritin concentration of more than 1000 ng/mL[13]. Because iron metabolism could be affected by anemia and iron overload, 1280 participants with anemia and iron overload were excluded from the study population.

### Statistical analysis

For the baseline characteristics of participants, all data are shown as the means±standard deviations (SDs) for normal variables and as medians (interquartile ranges) for skewed variables. Differences in characteristics across quartiles of serum ferritin for both gender groups, separately, were tested for significance. The unpaired t-test or Mann-Whitney U test was used to compare the differences between continuous variables, and the chi-square test was used for categorical variables. Bonferroni correction was made based on 10 different comparisons between metabolically healthy and metabolically abnormal groups, when necessary, thus, a two-tailed P value of 0.05 divided by 6, 0.008, was considered significant. Multivariable logistic regression analyses were performed to examine the association between different ferritin levels with dyslipidemia, lipid parameters and diabetes, HOMA-IR, separately. Because some data were skewed, we used quartile levels to analyze these data during the multivariable logistic regression analyses. The odds ratios (ORs) (95% CI) for dyslipidemia, lipid parameters and diabetes, HOMA-IR were calculated for quartiles of serum ferritin concentration, with the lowest ferritin quartile serving as the reference. The ORs were adjusted for age, BMI, smoking status, alcohol consumption, education level, systolic and diastolic blood pressure (SBP and DBP), CRP levels, daily Carbohydrate, fat, energy and protein intake, and further adjusted for parameters of glucose or lipid metabolism in the different regression models. The statistical analysis was performed with SAS 9.4 (SAS Institute, Cary, North Carolina).

## Results

### Baseline characteristics of the study subjects

The baseline characteristics are shown in Table 2. A total of 7109 participants (3522 men and 3587 women), aged 50.27±14.79 years (range, 18–97 years) with a BMI of 23.52±3.48 kg/m^2^, were included in this study. Based on quartiles of serum ferritin, participants were categorized into four subgroups in both gender groups. The men showed higher ferritin levels [123.28(132.64) vs. 53.50(67.33), P<0.001], SBP (125.94±17.43 vs. 123.92±20.10, P<0.001), DBP (82.32±11.07 vs. 79.87±11.49, P = 0.029), and TG levels [1.28(1.06) vs. 1.23(0.93), P = 0.004] and higher rates of current smoking (55.97% vs. 3.95%, P<0.001), alcohol consumption (61.01% vs. 8.97%, P<0.001) and higher carbohydrate intake [313.30(141.23) vs. 260.31(113.72), P<0.001], fat intake [74.25(46.11) vs. 64.62(41.48), P<0.001], energy intake [2286.30(848.25) vs. 1915.61(730.00), P<0.001] and protein intake [68.61(31.05) vs. 58.30(26.05), P<0.001] than the women. However, the men had lower TC levels [4.73(1.24) vs. 4.87(1.35), P<0.001], HDL-C [1.30(0.47) vs. 1.43(0.45), P<0.001] and LDL-C [2.88(1.15) vs. 2.99(1.26), P<0.001] than the women. Other demographic and clinical characteristics were not significantly different between the two genders.

**Table 2:** Baseline characteristics of the participants by sex-specific quartiles of serum ferritin.

	Men (3522)				P for trend^a^	Women (3587)				P for trend^a^	P value^b^
	Q1	Q2	Q3	Q4		Q1	Q2	Q3	Q4		
**Ferritin, mg/L**	56.42 (23.92)	100.06 (22.14)	154.27 (39.58)	379.90 (323.36)	<0.001	24.52 (9.39)	47.12 (12.39)	77.99 (20.83)	156.40 (109.31)	<0.001	<0.001
**Age, years**	51.43+15.52	49.81+15.21	49.67+14.80	49.02+13.66	0.002	40.78+11.60	47.12+14.35	54.12+13.65	60.22+11.32	<0.001	0.682
**BMI, kg/m2**	22.63+3.28	23.07+3.35	23.52+3.30	24.38+3.42	<0.001	23.06(3.50)	23.29(3.39)	23.82(3.57)	24.41(3.63)	<0.001	0.005
**SBP, mm Hg**	126.27+18.41	125.70+17.84	125.37+16.61	126.43+16.78	0.432	117.11+16.72	120.88+18.39	126.18+19.92	131.54+22.01	<0.001	<0.001
**DBP, mm Hg**	81.63+11.03	81.84+11.01	82.12+11.11	83.70+11.04	<0.001	77.23+10.76	78.32+10.70	80.90+11.42	83.03+12.12	<0.001	0.029
**TC, mmol/L**	4.62(1.24)	4.60(1.13)	4.79(1.17)	4.97(1.38)	<0.001	4.53(1.11)	4.70(1.24)	5.06(1.40)	5.26(1.32)	<0.001	<0.001
**TG, mmol/L**	1.06(0.75)	1.21(0.86)	1.34(1.11)	1.64(1.38)	<0.001	1.00(0.69)	1.13(0.82)	1.27(0.91)	1.57(1.19)	<0.001	0.004
**HDL-C, mmol/L**	1.38(0.45)	1.33(0.48)	1.29(0.46)	1.23(0.47)	<0.001	1.47(0.44)	1.44(0.44)	1.44(0.47)	1.38(0.45)	<0.001	<0.001
**LDL-C, mmol/L**	2.80(1.16)	2.85(1.02)	2.94(1.17)	2.98(1.36)	0.009	2.74(0.99)	2.88(1.18)	3.20(1.30)	3.28(1.40)	<0.001	<0.001
**FPG, mmol/L**	5.00(0.89)	5.04(0.94)	5.10(0.93)	5.20(1.09)	<0.001	4.96(0.72)	5.03(0.84)	5.10(0.95)	5.24(1.08)	<0.001	0.821
**HbA1c, %**	5.50(0.60)	5.50(0.60)	5.50(0.60)	5.60(0.60)	<0.001	5.40(0.50)	5.50(0.70)	5.50(0.60)	5.70(0.70)	<0.001	0.464
**Insulin, μIU/mL**	9.50(6.87)	10.19(7.43)	10.79(8.26)	11.50(9.57)	<0.001	10.08(7.19)	10.28(7.40)	11.02(7.14)	12.16(9.00)	<0.001	0.080
**HOMA-IR**	2.14(1.76)	2.31(1.87)	2.44(2.19)	2.68(2.84)	<0.001	2.22(1.67)	2.28(1.82)	2.49(1.93)	2.92(2.54)	<0.001	0.122
**Hemoglobin, g/L**	153.20+14.88	154.93+14.20	155.98+14.75	157.28+14.05	<0.001	136.52+14.49	137.63+14.03	138.46+15.08	138.36+14.39	<0.001	
**Smokers, %**	59.82	61.48	63.90	63.98	0.071	2.34	4.01	4.79	5.92	0.002	<0.001
**Alcohol use/last year, %**	55.96	58.75	65.61	63.75	<0.001	7.36	9.70	9.81	7.70	0.127	<0.001
**Education, %**					<0.001					<0.001	<0.001
**Low**	36.89	31.25	33.83	28.98		35.23	43.59	56.41	69.31		
**Medium**	39.61	38.86	36.78	40.34		41.92	31.77	23.86	17.97		
**High**	23.50	29.89	29.40	30.68		22.85	24.64	19.73	12.72		
**Daily carbohydrate intake, g**	316.32(149.92)	316.07(146.07)	315.96(135.11)	306.14(131.55)	0.190	266.53(116.46)	262.46(119.93)	260.25(118.52)	252.86(103.38)	0.007	<0.001
**Daily fat intake, g**	72.51(46.82)	74.46(44.87)	73.46(45.42)	76.83(47.98)	0.241	64.80(40.66)	64.22(39.30)	65.02(42.23)	64.59(45.90)	0.498	<0.001
**Daily energy intake, kcal**	2249.02(861.21)	2320.85(834.65)	2311.53(849.69)	2293.11(870.14)	0.476	1947.47(755.94)	1940.57(726.48)	1945.15(749.25)	1843.30(682.65)	0.029	<0.001
**Daily protein intake, g**	66.25(31.44)	68.16(29.80)	69.82(31.11)	70.90(30.61)	0.007	59.18(27.31)	58.87(24.87)	58.37(26.89)	57.26(25.55)	0.126	<0.001

Values are presented as the mean±SD or median (interquartile range) or percent

BMI, body mass index; SBP, systolic blood pressure; DBP, diastolic blood pressure; TC, total cholesterol; TG, triglycerides; HDL-C, high-density lipoprotein cholesterol; LDL-C, low-density lipoprotein cholesterol; FPG, fasting plasma glucose; HbA1c, glycated hemoglobin A1c; HOMA-IR, homeostatic model assessment of insulin resistance.

^a^ P for trend across quartiles of serum ferritin within gender group.

^b^ P for difference of both gender groups.

**Fig 1:**
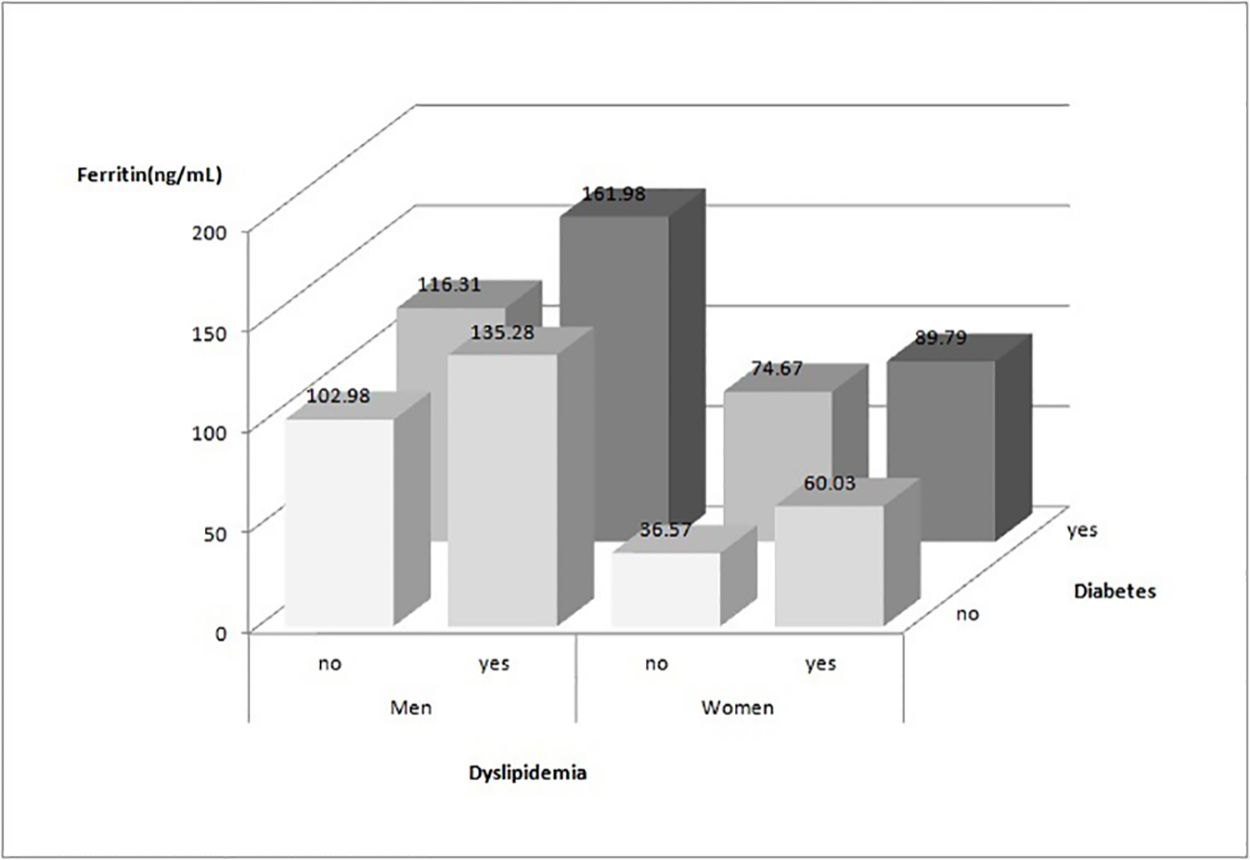
Serum ferritin levels in different gender groups according to dyslipidemia and diabetes status.

Serum ferritin levels in different gender groups according to dyslipidemia and diabetes status are shown in Fig 1. The men showed higher serum ferritin levels than the women in four subgroups. And subjects with dyslipidemia and diabetes had higher serum ferritin levels than subjects without dyslipidemia and diabetes in both gender groups (P<0.001). Subjects with dyslipidemia had higher serum ferritin levels than subjects with diabetes in men (135.28 vs. 116.31 ng/ml, P<0.001), but lower in women (60.03 vs. 74.67 ng/ml, P<0.001).

### Adjusted ORs and 95% CIs for dyslipidemia and lipid parameters according to sex-specific quartiles of serum ferritin

Table 3 shows the adjusted ORs and 95% Cls for dyslipidemia and lipid parameters, according to sex-specific quartiles of serum ferritin. For both genders, the ORs for the risk of dyslipidemia, high TG, high TC, high LDL-C, and low HDL-C levels increased and for HDL-C decreased progressively across the ferritin quartiles (P<0.001 for trend). After adjustment for age, BMI, smoking status, alcohol consumption, education level, SBP, DBP, CRP levels, daily carbohydrate intake, fat intake, energy intake and protein intake in the logistic regression model (Model 2), and further for history of diabetes and HOMA-IR in Model 3, the results remained unchanged.

**Table 3:** Adjusted odds ratio (95% CI) for dyslipidemia and lipid parameters by sex-specific quartiles of serum ferritin.

	Men (3522)				P value	Women (3587)				P value
	Q1	Q2	Q3	Q4		Q1	Q2	Q3	Q4	
**Ferritin (mg/L)**	56.42 (23.92)	100.06 (22.14)	154.27 (39.58)	379.90 (323.36)		24.52 (9.39)	47.12 (12.39)	77.99 (20.83)	156.40 (109.31)	
**Dyslipidemia**										
Model 1^a^	1	1.12(0.93–1.36)	1.59(1.32–1.93)	2.75(2.25–3.37)	<0.001	1	1.56(1.29–1.88)	2.64(2.16–3.23)	4.24(3.40–5.28)	<0.001
Model 2^b^	1	1.06(0.86–1.29)	1.41(1.15–1.73)	2.12(1.70–2.64)	<0.001	1	1.35(1.10–1.66)	1.79(1.43–2.24)	2.23(1.73–2.89)	<0.001
Model 3^c^	1	1.05(0.86–1.29)	1.37(1.12–1.69)	2.02(1.62–2.52)	<0.001	1	1.36(1.11–1.67)	1.84(1.46–2.31)	2.14(1.64–2.78)	<0.001
**TG**										
Model 1	1	1.40(1.12–1.75)	2.13(1.72–2.63)	3.68(2.99–4.52)	<0.001	1	1.51(1.19–1.90)	2.19(1.75–2.74)	4.02(3.24–4.99)	<0.001
Model 2	1	1.30(1.03–1.64)	1.88(1.50–2.35)	2.86(2.30–3.56)	<0.001	1	1.32(1.04–1.69)	1.59(1.24–2.03)	2.47(1.92–3.17)	<0.001
Model 3	1	1.27(1.00–1.60)	1.82(1.45–2.28)	2.66(2.13–3.32)	<0.001	1	1.32(1.03–1.69)	1.59(1.24–2.04)	2.37(1.84–3.06)	<0.001
**TC**										
Model 1	1	1.01(0.82–1.25)	1.43(1.17–1.74)	2.07(1.70–2.52)	<0.001	1	1.50(1.22–1.84)	2.77(2.27–3.39)	3.67(3.01–4.47)	<0.001
Model 2	1	0.98(0.79–1.21)	1.34(1.09–1.66)	1.77(1.44–2.17)	<0.001	1	1.17(0.94–1.46)	1.63(1.31–2.03)	1.68(1.34–2.11)	<0.001
Model 3	1	0.96(0.77–1.19)	1.31(1.06–1.62)	1.67(1.35–2.06)	<0.001	1	1.18(0.95–1.47)	1.65(1.33–2.06)	1.66(1.32–2.08)	<0.001
**HDL-C**										
Model 1	1	0.86(0.72–1.02)	0.74(0.63–0.88)	0.54(0.45–0.64)	<0.001	1	0.91(0.77–1.08)	0.93(0.78–1.10)	0.72(0.60–0.85)	0.001
Model 2	1	0.91(0.76–1.08)	0.88(0.73–1.05)	0.70(0.59–0.85)	0.002	1	0.88(0.74–1.05)	0.91(0.76–1.09)	0.72(0.59–0.88)	0.005
Model 3	1	0.91(0.76–1.09)	0.87(0.73–1.04)	0.70(0.58–0.85)	0.002	1	0.89(0.74–1.06)	0.90(0.75–1.09)	0.72(0.59–0.89)	0.016
**LDL-C**										
Model 1	1	0.86(0.69–1.07)	1.11(0.90–1.37)	1.37(1.12–1.68)	<0.001	1	1.67(1.34–2.08)	3.23(2.62–3.99)	3.63(2.94–4.48)	<0.001
Model 2	1	0.85(0.68–1.06)	1.08(0.87–1.33)	1.21(0.97–1.49)	0.014	1	1.30(1.03–1.63)	2.00(1.59–2.51)	1.72(1.36–2.51)	<0.001
Model 3	1	0.84(0.68–1.05)	1.06(0.86–1.32)	1.17(0.94–1.45)	0.031	1	1.30(1.03–1.65)	2.02(1.60–2.54)	1.69(1.32–2.15)	<0.001

TC, total cholesterol; TG, triglycerides; HDL-C, high-density lipoprotein cholesterol; LDL-C, low-density lipoprotein cholesterol;

^a^: Model 1, ORs without adjustment;

^b^ Model 2, ORs adjusted for Model 1 plus age, BMI, smoking status, alcohol consumption, education level, systolic and diastolic blood pressure, CRP levels, daily Carbohydrate, fat, energy and protein intake.

^c^ Model 3, ORs adjusted for Model 2 plus history of diabetes and HOMA-IR.

### Adjusted ORs and 95% CIs for the risk of diabetes and HOMA-IR according to sex-specific quartiles of serum ferritin

Table 4 shows the adjusted ORs and 95% Cls for the risk of diabetes and HOMA-IR, according to sex-specific quartiles of serum ferritin. The ORs for the risk of diabetes and high HOMA-IR level in the highest quartile group of serum ferritin levels were significantly increased in Model 1, but the association became weak or not significant after adjustment for lipid parameters in Model 3. The ORs for the risk of diabetes was decreased from 1.91 (95% CI: 1.37–2.67; P<0.001 for trend) to 1.48 (95% CI: 1.03–2.12; P = 0.036 for trend) in men, and from 5.40 (95% CI: 3.38–8.63; P<0.001 for trend) to 1.43 (95% CI: 0.83–2.43; P = 0.498 for trend) in women, and the ORs for IR was decreased from 1.86 (95% CI: 1.57–2.20; P<0.001 for trend) to 1.25 (95% CI: 1.05–1.50; P = 0.114 for trend) in men, and from 1.93 (95% CI: 1.63–2.28; P<0.001 for trend) to 1.24 (95% CI: 1.01–1.51; P = 0.012 for trend) in women.

**Table 4:** Adjusted odds ratio (95% CI) for the risk of diabetes and HOMA-IR by sex-specific quartiles of serum ferritin.

	Men (3522)				P value	Women (3587)				P value
	Q1	Q2	Q3	Q4		Q1	Q2	Q3	Q4	
**Ferritin (mg/L)**	56.42 (23.92)	100.06 (22.14)	154.27 (39.58)	379.90 (323.36)		24.52 (9.39)	47.12 (12.39)	77.99 (20.83)	156.40 (109.31)	
**diabetes**										
Model 1^a^	1	0.89(0.61–1.31)	1.32(0.93–1.88)	1.91(1.37–2.67)	<0.001	1	1.85(1.09–3.14)	3.05(1.86–5.00)	5.40(3.38–8.63)	<0.001
Model 2^b^	1	0.91(0.61–1.35)	1.33(0.92–1.92)	1.82(1.28–2.60)	<0.001	1	1.24(0.71–2.15)	1.40(0.82–2.39)	1.85(1.10–3.13)	0.063
Model 3^c^	1	0.89(0.60–1.32)	1.20(0.83–1.75)	1.48(1.03–2.12)	**0.036**	1	1.11(0.63–1.95)	1.22(0.71–2.09)	1.43(0.84–2.43)	**0.498**
**HOMA-IR**										
Model 1	1	1.19(1.01–1.41)	1.36(1.15–1.61)	1.86(1.57–2.20)	<0.001	1	0.99(0.84–1.18)	1.29(1.09–1.52)	1.93(1.63–2.28)	<0.001
Model 2	1	1.16(0.98–1.38)	1.23(1.03–1.46)	1.45(1.22–1.73)	<0.001	1	0.96(0.81–1.14)	1.09(0.91–1.31)	1.45(1.19–1.76)	<0.001
Model 3	1	1.11(0.94–1.32)	1.13(0.95–1.34)	1.25(1.05–1.50)	**0.114**	1	0.91(0.77–1.09)	0.99(0.82–1.19)	1.24(1.01–1.51)	**0.012**

HOMA-IR, homeostatic model assessment of insulin resistance.

^a^: Model 1, ORs without adjustment;

^b^ Model 2, ORs adjusted for Model 1 plus age, BMI, smoking status, alcohol consumption, education level, systolic and diastolic blood pressure, CRP levels, daily Carbohydrate, fat, energy and protein intake.

^c^ Model 3, ORs adjusted for Model 2 plus TC, TG, HDL-C and LDL-C levels.

## Discussion

This study found that elevated serum ferritin levels were associated with the prevalence of dyslipidemia among Chinese adults in a large cross-sectional study (2009 CHNS). There was a significant positive association between serum ferritin levels and lipid parameters independent of diabetes and IR in both genders. We speculated that the relationship between serum ferritin and lipid metabolism was a key factor in energy metabolism disorders and an important potential role of diabetes and IR.

Several previous studies have indicated that elevated levels of circulating ferritin are associated with disrupted glucose and lipid metabolism, which is observed in T2DM[14–17], gestational diabetes[18, 19], IR[20, 21], β-cell dysfunction[22], obesity[23–27], dyslipidemia[20], CVD[28, 29], and MetS[3, 30]. This study also showed that subjects with dyslipidemia and diabetes had higher serum ferritin levels than subjects without dyslipidemia and diabetes in both genders. But what is the role of serum ferritin in those disorders?

The interaction between glucose and lipid metabolism is complex. The exact molecular mechanism of iron-related pathology in diabetes and dyslipidemia is not clearly understood. Recent studies have shown that diabetic dyslipidemia may not only be the consequence of diabetes but may also disrupt glucose metabolism. The lipotoxic effects result in impaired insulin secretion and β-cell apoptosis and may contribute to the loss of β-cell function in the pathogenesis of T2DM [4].

In a case-cohort study, Jehn et al. [17] found that persons with T2DM have higher ferritin levels. But after adjustment for BMI and components of the MetS, the hazard ratio decreased from 1.74 (95% CI: 1.14–2.65; p-trend<0.001) to 0.81 (95% CI: 0.49–1.34; p-trend = 0.87). So they indicated that elevated ferritin may be just one of several metabolic abnormalities related to the underlying process that ultimately results in diabetes, rather than a causal factor for diabetes. Similar results were found in other studies (Table 1).

Elevated levels of triglycerides lead to elevated levels of free fatty acids which may induce insulin resistance and β-cell dysfunction. High-density lipoprotein (HDL) is also the central component of reverse cholesterol transport and mediate cholesterol efflux from many tissues. This may change the micro environment such that insulin sensitivity and insulin secretion improve. [2] So serum ferritin may affect glucose metabolism through lipid metabolism.

Recently, Kim et al.[20] reported that serum ferritin levels were significantly associated with major dyslipidemia parameters in Korean adolescents. Although this study didn’t rule out the influence of glucose metabolism, it revealed that elevated serum ferritin levels may be associated with disrupted lipid metabolism and may be an independent risk factor of CVD.

As an iron-storage protein, serum ferritin is used to evaluate the status of common diseases, such as iron-deficiency anemia [21, 27], and hereditary and acquired iron overload conditions, such as hereditary hemochromatosis, thalassemia, hemoglobinopathy and chronic transfusion therapy [31]. Serum ferritin is also recognized as an acute phase marker of inflammation. Some studies have explored whether elevated serum ferritin levels are associated with low-grade inflammation [32, 33], such as in chronic kidney disease [34], rheumatoid arthritis [35] and other autoimmune disorders [36]. These diseases exhibit low-grade inflammation and are mediated by oxidative stress, which is characterized by an imbalance between the generation of reactive oxygen species (ROS) and responses from the antioxidant defense system[37].

Aranda et al.[32] observed that higher circulating ferritin increased lipid peroxidation. Iron is a strong pro-oxidant and can cause cellular damage by producing ROS in different tissues of the body[38]. Elevated ferritin levels augment pro-inflammatory cytokines that may mediate the association of ferritin with dyslipidemia. Therefore, we excluded participants with anemia and iron overload, used inflammation-adjusted ferritin concentration as ferritin level and adjusted for CRP levels in the multivariable logistic regression analyses in our study. However, adjusting for the CRP level in the present study did not change the association between serum ferritin and lipid parameters, suggesting that low-grade inflammation may not explain the association between serum ferritin levels and dyslipidemia[39]. But the effect of inflammation via high ferritin level is not excluded completely and warranted to provide further evidence in future study.

Our study is the first to explore the independent association between serum ferritin levels and dyslipidemia in general Chinese adults. In the multivariable logistic regression analyses, there was a strong positive association between serum ferritin levels and lipid parameters independent of the risk of diabetes and IR levels in both genders, but associations of serum ferritin levels with diabetes and IR became weak or non-significant after adjustment for lipid parameters.

There are several limitations of this study. First, this cross-sectional analysis did not examine temporal changes in dyslipidemia because biomarker data were collected only in the 2009 round of the CHNS. Second, we were unable to distinguish type 1 from type 2 diabetes in our study. Third, we were also unable to access the dietary iron data and cannot provide an overall analysis of iron and dyslipidemia status or explore the correlation between dietary iron and dyslipidemia. So we adjusted diet intake (including carbohydrate, fat, energy and protein) in the logistic regression model. This can partially correct the effects of dietary iron and lipids intake. However, in further study, the more accurate dietary iron data of will be of importance. Fourth, serum insulin was detected using the radioimmunoassay method. This method measures insulin, c-peptide, proinsulin, and proinsulin intermediate metabolites and may get a relatively higher result than other methods, such as immunoturbidimetric immunoassay method. Last, although no information for medication of dyslipidemia, we found 161 participants with medication for diabetes (including oral medicine and insulin injection) and 287 participants with noncommunicable disease (including dyslipidemia) through questionnaire. We obtained the same results after exclusion of these participants.

In conclusion, our results provide evidence that serum ferritin levels are significantly associated with major lipid parameters independent of diabetes and IR. This association was a key factor in energy metabolism disorders and an important potential role of diabetes and IR. Further studies, especially well-designed observational and cohort studies and randomized trials, are warranted to provide stronger evidence and establish a causal inference.
